# Lack of awareness of treatment failure among HIV-1-infected patients in Guinea-Bissau – a retrospective cohort study

**DOI:** 10.7448/IAS.18.1.20243

**Published:** 2015-09-29

**Authors:** Sanne Jespersen, Bo Langhoff Hønge, Candida Medina, David da Silva Té, Faustino Gomes Correira, Alex Lund Laursen, Christian Erikstrup, Lars Østergaard, Christian Wejse, for the Bissau HIV Cohort study group

**Affiliations:** 1 Bandim Health Project, Indepth Network, Bissau, Guinea-Bissau; 2 Department of Infectious Diseases, Aarhus University Hospital, Aarhus, Denmark;; 3 Department of Clinical Immunology, Aarhus University Hospital, Aarhus, Denmark; 4 National HIV Programme, Ministry of Health, Bissau, Guinea-Bissau; 5 GloHAU, Center for Global Health, School of Public Health, Aarhus University, Aarhus, Denmark

**Keywords:** HIV, treatment failure, viral load, CD4 cell count, Guinea-Bissau, Africa

## Abstract

**Introduction:**

With more people receiving antiretroviral treatment (ART), the need to detect treatment failure and switch to second-line ART has also increased. We assessed CD4 cell counts (as a marker of treatment failure), determined the rate of switching to second-line treatment and evaluated mortality related to treatment failure among HIV-infected patients in Guinea-Bissau.

**Methods:**

In this retrospective cohort study, adult patients infected with HIV-1 receiving ≥6 months of ART at an HIV clinic in Bissau were included from June 2005 to July 2014 and followed until January 2015. Treatment failure was defined as 1) a fall in CD4 count to baseline (or below) or 2) CD4 levels persistently below 100 cells/µL after ≥6 months of ART. Cox hazard models, with time since six months of ART as the time-varying coefficient, were used to estimate the hazard ratio for death and loss to follow-up.

**Results:**

We assessed 1,591 HIV-1-infected patients for immunological treatment failure. Treatment failure could not be determined in 594 patients (37.3%) because of missing CD4 cell counts. Among the remaining 997 patients, 393 (39.4%) experienced failure. Only 39 patients (9.9%) with failure were switched from first- to second-line ART. The overall switching rate was 3.1 per 100 person-years. Mortality rate was higher in patients with than without treatment failure, with adjusted hazard rate ratios (HRRs) 10.0 (95% CI: 0.9–107.8), 7.6 (95% CI: 1.6–35.5) and 3.1 (95% CI: 1.5–6.3) in the first, second and following years, respectively. During the first year of follow-up, patients experiencing treatment failure had a higher risk of being lost to follow-up than patients not experiencing treatment failure (adjusted HRR 4.4; 95% CI: 1.7–11.8).

**Conclusions:**

We found a high rate of treatment failure, an alarmingly high number of patients for whom treatment failure could not be assessed, and a low rate of switching to a second-line therapy. These factors could lead to an increased risk of resistance development and excess mortality.

## Introduction

With the rapid scale-up of antiretroviral treatment (ART) availability in sub-Saharan Africa, the need for appropriate treatment monitoring has also increased [1]. As more people receive ART, more will experience treatment failure and need to switch to second-line ART in resource-limited settings. The World Health Organization (WHO) recommends viral load (VL) as the preferred monitoring approach to diagnose and confirm ART failure; however, if VL is not routinely available, CD4 cell count and clinical monitoring are recommended instead [2]. The main rationale for recommending VL monitoring as the preferred approach is to obtain an early and more accurate indication of treatment failure and the need to switch to second-line drugs, thereby reducing the accumulation of drug-resistant mutations and improving clinical outcomes [2]. Unfortunately, VL monitoring is still not available in many parts of Africa, leaving the clinician unaware of treatment failure and increasing the risk of developing resistance [3].

In accordance with WHO guidelines, most HIV-1-infected patients in Africa initiate ART with two nucleoside/nucleotide reverse transcriptase inhibitors (NRTIs) and one non-nucleotide reverse transcriptase inhibitor (NNRTI), with the NNRTI being either nevirapine (NVP) or efavirenz (EFV) [4].

In Bissau, the capital of the West African country Guinea-Bissau, the prevalence of HIV-1 infection has been increasing (4.4% in 2006), and the prevalence of HIV-2 (4.4%) is the highest of any country in the world. A small proportion of individuals are dually infected with both HIV-1 and HIV-2 (0.5%) [5,6]. A range of persistent problems affect feasible ART administration in Guinea-Bissau, including intermittent drug supplies, poor adherence and patient retention, and inadequate laboratory facilities [7].

The aim of this study was (1) to assess immunologic failure rates in patients completing six or more months of ART, (2) to determine the rate of treatment switching in patients with or without immunologic failure and (3) to assess the mortality rate of patients, with or without treatment failure.

## Methods

### Setting and study population

We included patients from the HIV clinic at the Hospital National Simão Mendes (HNSM) in Bissau. The clinic is the base of the Bissau HIV Cohort, and the study aims and characteristics of the cohort have been described in detail previously [8]. The study population consisted of HIV-1 mono-infected adults who were diagnosed at HNSM and whose ART was initiated between June 2005, when the clinic opened, and July 2014. According to WHO guidelines, an individual must be taking ART for at least six months before it can be determined that a regimen has failed [2]. We, therefore, included all HIV-1-infected patients who remained in care after at least six months of ART.

### Data collection

At the first visit to the clinic, HIV testing was performed and, if the result was positive, basic demographic information was collected and patients were given a requisition for laboratory analyses (CD4 cell count, biochemistry and haematology). Blood sampling was usually performed at the clinic on the following day. Patients receiving ART were normally seen on a monthly or bimonthly basis and were scheduled to have a CD4 cell count performed every six months. VL measurements were not available in Guinea-Bissau during the study period. Patients on ART were considered lost to follow-up (LTFU) if they had not visited the clinic for six months. Information on death and transfer to other ART centres was collected by personal information, telephone calls with contact persons or from the hospital wards [9].

### ART guidelines

Patients were eligible for ART in accordance with WHO guidelines [4]. At the time of this study, the standard first-line ART regimen for HIV-1-infected patients consisted of two NRTIs (zidovudine [AZT] and lamivudine [3TC]) and one NNRTI (either NVP or EFV) with the substitution of AZT for stavudine (D4T) or abacavir in case of anaemia. In January 2012, AZT was replaced with tenofovir (TDF) as the preferred NRTI in patients with anaemia or hepatitis B co-infection. Some patients initiated treatment with a protease inhibitor (PI)-based first-line treatment regimen as part of a randomized controlled trial ongoing at the clinic comparing an NNRTI with a PI-based treatment regimen (clinicaltrials.gov identifier: NCT0019235). In accordance with the local guidelines, patients should be switched to second-line treatment based on clinical and/or immunological criteria [4]. Furthermore, patients could be switched to second-line therapy if the drug was out of stock or if the patient experienced adverse events. Second-line therapy in this study is defined as a switch from NNRTI to PI or triple NRTI, a switch from PI to NNRTI or triple NRTI or a switch from triple NRTI to NNRTI or PI. The PIs available include ritonavir-boosted lopinavir (LPV/r) and ritonavir-boosted indinavir (IDV/r). ART and laboratory analyses are free of charge to all HIV-infected patients in Guinea-Bissau. The ART regimen for each patient was assessed at initiation of treatment, at switch to second-line therapy and at the end of the study.

### Definition of treatment failure

In this study, treatment failure was based on immunological markers defined according to the WHO 2013 guidelines [10] as follows, (1) fall in CD4 counts to baseline (or below) or (2) CD4 levels persistently below 100 cells/µL. WHO recommends that CD4 cell counts are measured in patients every 6–12 months while they are on ART [2]. Baseline CD4 cell count before starting ART was compared to CD4 cell counts after at least six months of ART.

### Blood analyses

HIV screening was conducted with a rapid test (Determine HIV-1/2 assay, Abbott Laboratories, Abbott Park, IL, USA), and confirmation and discrimination were performed using the SD Bioline HIV 1/2 3.0 rapid test (Standard Diagnostics Inc., Kyonggi-do, South Korea). During 2012, the SD Bioline HIV 1/2 3.0 was gradually replaced by the First Response HIV card 1-2.0 (PMC Medical, Mumbai, India). CD4 cell counts were measured by flow cytometry using Partec CyFlow^®^ SL_3 (Cyflow SL, Partec, Munster, Germany).

### Statistical methods

Baseline characteristics of patients with and without treatment failure and those with unassessable treatment failure were compared using the chi-square test for categorical variables and the Kruskal–Wallis test for continuous variables (non-normal distribution). Median and interquartile range (IQR) were determined for continuous variables. The rate of treatment failure was determined by following patients from six months of ART exposure until the development of treatment failure. Cox hazard models, with time since six months of ART as the time-varying coefficient, were used to estimate the hazard ratio for death and loss to follow-up. The use of a time-varying coefficient allowed us to make comparisons among patients alive after 1, 2 or ≥3 years, respectively. The Cox model was adjusted for potential confounders (age, sex, baseline CD4 cell count and education). Differences in mortality rate ratio over time were compared with the Wald test.

Patients were followed from the date of six months of ART and until the patient's death, transfer to another clinic, LTFU or end of the study. Patients LTFU were censored at their last visit at the clinic. Patients who transferred to other ART centres were censored at the date of transfer. Other patients were censored on January 1, 2015, when all participants had been followed for at least six months. All statistical analyses were carried out using Stata IC 13.1 (StataCorp, College Station, TX, USA).

### Ethics

Prior to enrolment, all patients in the Bissau HIV Cohort voluntarily provided signed and dated informed consent or a fingerprint if they were unable to read or sign. We received approval to use data from patient files as long as patient confidentiality was respected. The study was approved by the UCEPS, the National Ethics Committee of Guinea-Bissau (Parecer NCP/No.15/2007).

## Results

### Baseline characteristics

**Figure 1:**
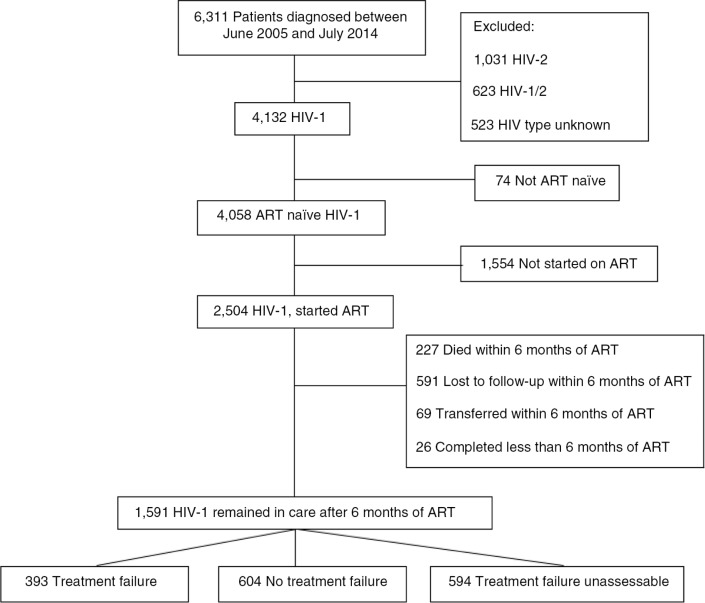
Flowchart of patients in the study. **ART=antiretroviral treatment**.

By July 1, 2014, a total of 6,311 HIV-infected adults had attended the clinic (4,132 HIV-1; 1,031 HIV-2; 623 HIV-1/2 dually infected and 525 patients with unknown HIV type). Only HIV-1 mono-infected patients were included in this study. Patients already receiving ART at enrolment were excluded (74 patients), as were patients who did not start ART during the study period (1,554 patients). Furthermore, patients not retained in care after at least six months of ART were excluded, leaving 1,591 patients for further analyses, representing 64% of all HIV-1-infected patients who initiated ART for the first time at the clinic (Figure 1).

### Initial treatment

Median time from HIV diagnosis at the clinic until the start of ART was 17 days (IQR: 8–57 days). The majority of patients (1383/1591, 87%) were started on NNRTI-based treatment regimens while 194 (12%) and 13 patients (1%) were started on PI-based and triple NRTI regimens, respectively. The most common ART combinations were AZT/3TC/NVP (40%), AZT/3TC/EFV (22%), TDF/FTC/EFV (12%), D4T/3TC/NVP (7%) and AZT/3TC/LPV/r (6%).

### Immunological treatment failure

Among 1,591 patients who remained in care after at least six months of treatment, 997 patients (63%) had a CD4 cell count measurement before and at least six months after starting ART, making it possible to assess immunological treatment failure (Figure 1). Treatment failure could not be assessed in 594 patients (37%). Baseline CD4 cell count was measured an average of 12 days before starting ART (IQR: 7–28 days).

Patients were followed from having received six months of ART and contributed to 3,850 person-years of observation. The median number of CD4 cell count measurements after receiving six months of ART was 2 (IQR: 1–4). With a median follow-up time of 34 months (IQR: 21–56 months), patients had a CD4 cell count measurement every fifteenth month (IQR: 11–21 months), with the most frequent measurements done in patients experiencing treatment failure (Table 1). The median time from the start of ART until the first CD4 cell count measurement that could be used to assess treatment failure was 10 months (8–15 months).

**Table 1:** Demographic and clinical characteristics

		Total *n=*1591	Treatment failure *n=*393	No treatment failure *n=*604	Treatment failure unassessable *n=*594	*p*
Sex (%)						0.011
	Females	1043 (65.5)	264 (67.2)	416 (68.9)	362 (60.9)	
	Males	549 (34.5)	129 (33.8)	188 (31.1)	232 (39.1)	
Age, median years (IQR)		35 (29–43)	34 (29–42)	35 (29–42)	35 (30–44)	0.218
Age strata, n (%)						0.185
	18–29 years	426 (26.8)	107 (27.2)	173 (28.6)	146 (24.6)	
	30–36 years	452 (28.4)	119 (30.3)	167 (27.7)	166 (28.0)	
	37–45 years	429 (30.0)	95 (24.2)	172 (28.5)	162 (27.3)	
	≥46 years	282 (17.8)	72 (18.3)	92 (15.2)	118 (19.9)	
	Missing	2 (0.1)	0 (0)	0 (0)	2 (0.3)	
Baseline CD4 before ART, median cells/µl (IQR)		169 (83–254)	188 (72–271)	160 (88–239)	159 (82–255)	0.141
CD4 cell count strata, n (%)						<0.001
	≤100 cells/µl	367 (23.1)	118 (30.0)	179 (29.6)	70 (11.8)	
	101–200 cells/µl	362 (22.8)	91 (23.2)	202 (33.4)	69 (11.6)	
	201–350 cells/µl	420 (26.4)	153 (39.0)	199 (33.0)	68 (11.5)	
	>350 cells/µl	80 (50.0)	31 (7.9)	24 (4.0)	25 (4.2)	
	Missing	362 (22.8)	0 (0)	0 (0)	362 (60.9)	
Time between CD4 cell count measurements after six months of ART, median months (IQR)		15 (11–21)	15 (11–21)	14 (10–21)	16 (12–22)	0.011
Marital status, n (%)						0.748
	Married	823 (51.7)	215 (54.7)	296 (49.0)	312 (52.5)	
	Divorced	108 (6.8)	28 (7.1)	44 (7.3)	36 (6.2)	
	Widowed	79 (13.3)	53 (13.5)	82 (13.6)	79 (13.3)	
	Single	160 (27.0)	94 (23.9)	175 (29.0)	160 (26.9)	
	Missing	17 (1.1)	3 (0.8)	7 (1.2)	7 (1.2)	
Education, n (%)						0.014
	None	449 (28.2)	127 (32.3)	174 (28.8)	148 (24.9)	
	1–4 years	157 (9.9)	38 (9.7)	54 (8.9)	65 (10.9)	
	5–11 years	890 (55.9)	205 (52.2)	352 (58.3)	333 (56.1)	
	Missing	95 (6.0)	23 (5.9)	24 (4.0)	48 (8.1)	

Numbers may not add up to 100% because of rounding errors. ART=antiretroviral treatment; IQR=interquartile range.

Immunological treatment failure was detected in 393 patients (24.7%). For 183 patients (11.5%), failure was detected based on CD4 cell counts persistently below 100 cells/µL, whereas 210 patients (13.2%) were classified as having treatment failure because their most recent CD4 cell count was lower than their baseline CD4 cell count before receiving ART. The rate of treatment failure among patients remaining in care after at least six months of ART was 20.3 per 100 person-years (95% confidence interval [CI]: 18.4–22.4) among patients for whom treatment failure was assessable. After exclusion of patients not starting on an NNRTI-based treatment regimen, the rate of treatment failure was 19.6 per 100 person-years (95% CI: 11.6–33.2). The characteristics of these patients are presented in Table 1.

### Switching to second-line treatment

A total of 127/1591 patients (8.0%) were switched to second-line treatment during the follow-up period. Reasons for switching to second-line treatment included treatment failure (55/127, 43.3%), ART out of stock (40/127, 31.5%), reason not specified (23/127, 18.1%), interaction with tuberculosis therapy (5/127, 3.9%) and adverse events (4/127, 3.2%). Of 1,383 patients, 58 (4.2%) treated with NNRTIs switched to PIs while 26 (1.9%) switched to triple NRTIs. Of 194 patients, 17 (8.8%) treated with PIs switched to NNRTIs, and 16 (8.3%) switched to triple NRTIs. Among 13 patients initially treated with triple NRTIs, four (30.8%) and six (46.2%) switched to NNRTIs or PIs, respectively.

Of the 127 patients, 36 (28.3%) later switched their ART drugs again before the end of the study. This group mainly consisted of patients switched back to their initial ART after a period during which this particular ART was out of stock but later became available again.

Only 39 (9.9%) of the 393 patients who experienced immunological treatment failure were in the group of patients who were switched to second-line therapy whereas 51 (8.6%) of the 594 patients for whom immunological treatment failure could not be assessed were switched. The rate of switching to second-line therapy because of immunological failure as defined in this study was 3.1 per 100 person-years (95% CI: 2.2–4.4). Because some patients were switched from an NNRTI to a PI, whether they had treatment failure or not, the overall switch rate was 2.7 per 100 person-years (95% CI: 2.2–3.3).

### Mortality and loss to follow-up

Of 1591 patients, 125 (7.9%) died, 71 patients (4.5%) were transferred to other ART centres and 478 were LTFU (30.0%), leaving 917 patients (57.6%) alive during follow-up at the end of this study. Fifty-five of the deaths occurred among patients for whom treatment failure was assessable. Mortality during the first, second and following years of follow-up in the study is presented in Table 2. The mortality rate was higher among patients who experienced treatment failure than in those without treatment failure. There was no significant difference in the mortality rate ratio over time (*p=*0.42).

**Table 2:** Mortality among patients with and without treatment failure

Year of follow-up		MR (D/PYO)	MRR (95% CI)	Adjusted MRR (95% CI)
First year				
	No treatment failure	13.5 (2/15)	1	1
	Treatment failure	47.6 (2/4)	3.9 (0.5–28.6)	10.0 (0.9–107.8)
Second year				
	No treatment failure	1.9 (2/107)	1	1
	Treatment failure	21.7 (13/60)	9.8 (2.2–43.7)	7.6 (1.6–35.5)
Third and following years				
	No treatment failure	0.8 (11/1328)	1	1
	Treatment failure	2.4 (25/1038)	2.8 (1.4–5.7)	3.1 (1.5–6.3)

Adjusted for age, sex, initial CD4 cell count and education. MR=mortality rate per 100 person-years of observation; D=deaths; PYO=person-years of observation, MRR=mortality rate ratios. CI=confidence interval.

During the first year of follow-up, patients experiencing treatment failure had a higher risk of being LTFU than patients not experiencing treatment failure (adjusted hazard rate ratio 4.4 [95% CI: 1.7–11.8]).

## Discussion

The three major findings of this study are that 1) one third of all patients were not assessed for treatment failure, 2) one third of those who were assessed experienced treatment failure and 3) only one in ten patients experiencing treatment failure were switched to second-line treatment. Consequently, patients who experienced treatment failure had increased mortality.

The strength of this study is the inclusion of all HIV-1-infected patients attending the largest HIV clinic in Guinea-Bissau. Furthermore, the study represents the real-life situation of one of the poorest countries in the world. This study is, however, limited by lack of data on adherence and clinical outcome. Treatment failure, whether immunological or virological, should be confirmed with a second measurement after assessing adherence, which was not the case in our study. This gap could have led to an overestimation of the true prevalence of treatment failure. On the other hand, it was not possible to assess treatment failure because CD4 cell count measurements were lacking in more than one third of patients. This lack probably led to an underestimation of the prevalence of treatment failure since patients for whom failure was not assessable may be a subgroup with poor healthcare-seeking behaviour. The HIV clinic in Bissau has a high rate of LTFU [9], and mortality may have been underestimated because some of the patients classified as LTFU may have in fact died [11].

In the present study, we included only HIV-1-infected patients. We have previously shown that the SD Bioline HIV 1/2 3.0 rapid test is not optimal for discriminating HIV types [12,13]. However, although 5% of HIV-1-infected patients were incorrectly diagnosed with HIV-2 or HIV-1/2 dual infection, no HIV-2 and 13% of dually infected patients were misclassified as HIV-1. Thus, even though some patients may have experienced ART failure because they were infected with HIV-2, the effect on the current results is likely to be minimal. Whether HIV-2 infection inhibits HIV-1 disease progression is also debatable. Studies from Guinea-Bissau have supported this hypothesis [14,15] whereas results of other studies have not [16].

### Immunological treatment failure

Different definitions of immunological treatment failure have been proposed by the WHO, making comparisons between studies difficult [4,10]. In a study in Kenya, 5.7% had immunologically defined treatment failure after at least 12 months of ART [17]. The lower risk of treatment failure was found, despite looser definitions of failure according to the WHO 2006 guidelines in which a 50% fall in CD4 cell counts from the on-treatment peak value was also classified as treatment failure. An analysis of 10 treatment programmes in Africa and South America found an immunological failure rate of 3.3 per 100 person-years [18], which was lower than in our study (20.3 per 100 person-years). Studies of ART failure in the neighbouring countries of Senegal and Gambia focused primarily on virological failure [19–21]. The value of VL testing as a more sensitive and early indicator of treatment failure is increasingly recognized, and it is the gold standard for monitoring the response to ART [2,22,23]. WHO recommends VL testing as the preferred monitoring approach to diagnose and confirm treatment failure [2]. A systematic review found that current WHO clinical and immunological criteria have low sensitivity and low positive predictive value for identifying individuals with virological failure [24]; however, an evaluation from resource-limited countries found no evidence of improved mortality, reduced clinical failure or loss of treatment options when compared to programmes with VL testing [25,26].

The high rate of treatment failure in our study could be explained by a low adherence among patients at the clinic and was not affected by whether the patients received a PI or a NNRTI-based treatment regimen. A cross-sectional study among patients at the clinic showed that only 16% had remembered to take more than 80% or more of their medicine during the last month [27], which may be one reason for the high level of resistance previously reported for this HIV cohort [28].

Among patients in this study, 37.7% had no CD4 cell count measurement before initiating ART or after at least six months of ART. Because of an unstable reagent supply and machine breakdowns, CD4 cell count measurements were not available for patients at several time points during the study period. Furthermore, Guinea-Bissau has been considered politically unstable for many years, and coup attempts have occasionally closed down the HIV clinic and the laboratory. All patients should have access to CD4 cell count testing to optimize ART management and reduce disease progression and mortality [2,29].

### Switching to second-line therapies

An analysis of 11 ART programmes in sub-Saharan Africa found that the rate of switching was 2.2 per 100 person-years [30]. The incidence of switching to second-line regimens was 4.9 per 100 person-years in a retrospective analysis of a cohort of adults initiating a standard first-line ART at five public sector sites in three African countries [31]; the switching rate for adults followed by Médicins sans Frontières was 4.2 per 1000 person-years in sub-Saharan projects [32]. Our switching rate of 3.1 per 100 person-years is an overestimation of patients who switched due to treatment failure because more than half of the patients were switched for reasons other than treatment failure, that is, drug availability. Furthermore, we did not count in our analyses those patients who did not achieve six months of follow-up.

Patients who switched treatment in our study probably reflect only the most obvious cases of treatment failure because of our inability to accurately determine when a change in treatment regimen was indicated, given that VL testing is not available. The low rate of switching, even among patients in whom immunological treatment failure was detected, calls for an increased awareness of treatment failure. It may also indicate that ART programmes in many low-resource settings with similar conditions suffer from a lack of optimal care, in particular but not limited to the lack of availability of VL testing. Clinicians may doubt the immediate benefit of second-line therapy because of the higher cost, difficulties in ensuring patient treatment adherence due to the higher pill burden of PI-based regimens, absence of fixed-dose drug combinations and the fear that no further treatment options will be available if subsequent failure on second-line regimens occurs [33,34]. We have not assessed the reasons for the limited switch to second-line in our cohort, but the difficult access to updated CD4 cell counts and no access to VL may have had influence. In addition, it is a common perception among clinicians that treatment failure most likely is because of low adherence, as is also frequently occurring [27].

### Mortality

Few data are available on the mortality of patients who meet the WHO criteria for treatment failure but do not switch to second-line treatment. However, an analysis of 11 ART programmes in sub-Saharan Africa showed that the adjusted mortality rate ratios were 1.64 (95% CI: 0.84–3.22) in patients who switched and 3.29 (95% CI: 1.85–5.84) in patients failing first-line therapy compared with patients without treatment failure who remained on a first-line regimen [30]. These values are comparable to the results in our study for patients failing treatment when only a few patients switched to second-line therapy. Patient status as immunologic failure or non-failure is not determined when the follow-up time begins, making comparison of mortality rates between the two groups challenging.

A limitation of this analysis is that the assessment of treatment failure depends on the availability of measurements, which is dependent on healthcare-seeking behaviour and a clinical evaluation of the need for a CD4 measurement. There could be a higher chance of a patient being classified as experiencing treatment failure if the patient comes to the clinic frequently because treatment failure would be more likely to be detected in those who had more CD4 cell count measurements. However, this factor could lead to both under- and overestimation of the mortality rate among the treatment failure group. Patients failing treatment were more often LTFU, and a large proportion of patients LTFU may have died [35]; thus, the mortality rate among patients with treatment failure may in fact have been even higher if assessment of mortality had been possible in all patients LTFU.

Our results highlight the need for better methods to detect treatment failure and a stronger focus on switching patients with treatment failure to second-line treatments in Guinea-Bissau. There are probably many reasons for the lack of awareness of treatment failure, including problems with the delivery of ART because of inadequate drug supplies, loss to follow-up, lack of healthcare workers and inadequate laboratory services [7]. This lack of awareness can put patient lives in jeopardy. Some of these issues such as drug stockouts have improved over the long inclusion period of this study whereas others are still challenging for the delivery of ART in Guinea-Bissau. The need is urgent to understand why many patients who are experiencing treatment failure in sub-Saharan Africa remain on first-line ART. Awareness of treatment failure may be improved by introducing point-of-care VL testing, electronic decision-support systems, immediate ART initiation, mobile phone appointment reminders and non-cash financial incentives for linkage and retention [36,37].

## Conclusions

In conclusion, we identified a high rate of treatment failure, but an even more alarmingly high number of patients for whom treatment failure could not be assessed and patients experiencing treatment failure who continued their first-line regimen. This scenario could lead to an increased risk of resistance development and excess mortality.
